# Application of SWATH mass spectrometry in the identification of circulating proteins does not predict future weight gain in early psychosis

**DOI:** 10.1186/s12014-020-09299-2

**Published:** 2020-10-27

**Authors:** Adrian Heald, Narges Azadbakht, Bethany Geary, Silke Conen, Helene Fachim, Dave Chi Hoo Lee, Nophar Geifman, Sanam Farman, Oliver Howes, Anthony Whetton, Bill Deakin

**Affiliations:** 1 Department of Endocrinology, Salford Royal Hospital, Manchester, UK; 2 Faculty of Biology, Medicine and Health and Manchester Academic Health Sciences Centre, The University of Manchester, Manchester, UK; 3 Division of Informatics, Imaging and Data Sciences, The University of Manchester, Manchester, UK; 4 Stoller Biomarker Discovery Centre, The University of Manchester, Manchester, UK; 5 Division of Medical Education, The University of Manchester, Manchester, UK; 6 The Manchester Molecular Pathology Innovation Centre, The University of Manchester, Manchester, UK; 7 Mersey Deanery Psychiatry Training Rotation, Manchester, UK; 8 IOP, Psychosis Studies, London, UK; 9 Division of Neuroscience and Experimental Psychology, The University of Manchester, Manchester, UK; 10 Department of Diabetes and Endocrinology, Salford Royal Hospital, Salford, M6 8HD UK

**Keywords:** Schizophrenia, Molecular approaches, SWATH MS, Weight change

## Abstract

Weight gain is a common consequence of treatment with antipsychotic drugs in early psychosis, leading to further morbidity and poor treatment adherence. Identifying tools that can predict weight change in early psychosis may contribute to better-individualised treatment and adherence. Recently we showed that proteomic profiling with sequential window acquisition of all theoretical fragment ion spectra (SWATH) mass spectrometry (MS) can identify individuals with pre-diabetes more likely to experience weight change in relation to lifestyle change. We investigated whether baseline proteomic profiles predicted weight change over time using data from the BeneMin clinical trial of the anti-inflammatory antibiotic, minocycline, versus placebo. Expression levels for 844 proteins were determined by SWATH proteomics in 83 people (60 men and 23 women). Hierarchical clustering analysis and principal component analysis of baseline proteomics data did not reveal distinct separation between the proteome profiles of participants in different weight change categories. However, individuals with the highest weight loss had higher Positive and Negative Syndrome Scale (PANSS) scores. Our findings imply that mode of treatment i.e. the pharmacological intervention for psychosis may be the determining factor in weight change after diagnosis, rather than predisposing proteomic dynamics.

## Relevance statement

The matter of weight increase in the first years after diagnosis with psychosis is a huge concern for all involved health care professionals. We have here shown that proteomic profile does not relate to subsequent weight change for those diagnosed up to 5 years previously.

A study targeting first episode psychosis exclusively may provide more definitive results. Proteomics remains a potential avenue of research given that it can reveal clues as to underlying biological pathways and mechanisms.

## Introduction

Weight gain remains a major problem in people with schizophrenia and other major mental illnesses, with immediate and long-term consequences for cardiometabolic health (summarised in Vancampfort [1]).

In the United Kingdom, resources are increasingly being directed towards comorbidities especially in primary care, so a greater understanding of the health profile of schizophrenia individuals is essential, given that life expectancy is up to 17 years less for people with schizophrenia than for the background population in individuals with schizophrenia and other forms of severe enduring mental illness (SMI) [2].

First-generation antipsychotic (FGA) and second-generation antipsychotic (SGA) medications are used to treat schizophrenia. FGAs, also known as “typical antipsychotics,” mainly act via dopamine D2 receptor antagonism. SGAs, also known as “atypical antipsychotics”, are predominantly serotonin 5HT2A receptor antagonists in addition to D2 receptor antagonism [3]. Both classes of antipsychotic agents have been associated with weight gain [4].

Proteomic profiling is an important technique because it enables the identification of new biomarkers and new pathways to increase our understanding of the biological processes that may predispose to weight gain in people treated for schizophrenia.

In a group of individuals with impaired glucose regulation with no reported history of mental illness, we recently showed that proteomic profiling with sequential window acquisition of all theoretical fragment ion spectra (SWATH) mass spectrometry (MS) can identify individuals more likely to experience weight change in relation to changes in treatment or lifestyle [5]. No previous study has looked at how proteomic profiling may help to identify those more or less likely to put on weight in relation to treatment of psychosis.

We here investigated whether baseline proteomic profiles predicted weight change over a follow-up period of just over 12 months using data from the BeneMin clinical trial [6] of the anti-inflammatory antibiotic, minocycline, versus placebo combined with anti-psychotic treatment in people with recent onset psychosis related to baseline proteomic profile.

## Methods

In the BeneMin prospective study [6], 207 participants with relatively recent-onset schizophrenia (duration up to 5 years since diagnosis) were randomly assigned according to an automated permuted blocks algorithm, to receive minocycline (200 mg per day for 2 weeks, then 300 mg per day for the remainder of the follow-up period) or matching placebo, which were added to their continuing usual treatment. The primary clinical outcome was assessed using the Positive and Negative Syndrome Scales (PANSS) [7] at 2, 6, 9, and just over 12 months follow-ups after the baseline assessment. PANSS entails 30 items divided across three scales, positive scale (7 items), negative scale (7 items), and general psychopathology scale (16 items) that are used to assess and record the severity of positive symptoms, negative symptoms and general psychopathology symptoms in patients affected by schizophrenia. The absence of each assessed symptom is denoted as 1 and its extreme severity is denoted as 7. Total PANSS score is subsequently calculated by adding the scores from the three scales [7].

Participants also completed the Calgary Depression Scale for Schizophrenia (CDSS) [8], Global Assessment of Functioning (GAF) [9], the short Wechsler Adult Intelligence Scale-III (WAIS-III) measure of current Intelligence Quotient (IQ) for patients affected by schizophrenia [10] and an assessment of premorbid IQ using Wechsler Test of Adult Reading (WTAR) [11]. Inclusion and exclusion criteria are given in the original BeneMin paper [6].

Full ethics approval was obtained for this study. The North West Manchester Research Ethics Committee (reference number 11/NW/0218) approved the study. All participants gave informed consent.

From the BeneMin study participants with measured baseline proteomic profiles, weight and body mass index (BMI) were available for 83 participants at baseline and follow-up. There were 60 men and 23 women. Mean age was 25.7 (standard deviation 5.4) years. Of the 83 participants, 39 received minocycline and 44 received placebo (Table 1). Only one of the participants (with 30 kg weight gain at follow-up) received metformin in the course of the study. Metformin has previously been shown to facilitate weight reduction in people with schizophrenia [12].

**Table 1:** Statistical comparison of demographic and clinical variables between the four weight change categories

	Weight change category	Weight change category	Weight change category	Weight change category	Follow-up weight change cohort with SWATH MS data	P-value
	Loss >= 10 kg	Loss >= 3 kg AND < 10 kg	Within 3 kg	Gain >= 3 kg	Cohort	Comparison between 4 categories
Number of participants	4	18	23	38	83	
Number of days of follow-up since Baseline	391.0 ± 37.0	376.3 ± 18.6	385.5 ± 29.8	388.7 ± 29.8	385.2 ± 28.0	0.563
Missing	0	0	1	0	1	
Weight change at follow-up since Baseline (kg)	− 20.4 ± 9.8	− 5.8 ± 2.0	0.4 ± 1.6	10.9 ± 7.8	2.9 ± 10.3	**1.9e–15**
Missing	0	0	0	0	0	
Estimated duration of illness at Baseline (months)	30.0 ± 26.9	13.8 ± 9.3	22.7 ± 18.7	18.6 ± 18.7	19.3 ± 17.4	0.595
Missing	2	6	5	12	25	
BeneMin treatment allocation						0.852
Minocycline	1	8	11	19	39	
Placebo	3	10	12	19	44	
Missing	0	0	0	0	0	
Age at baseline (years)	31.0 ± 5.9	27.0 ± 6.1	23.9 ± 3.7	25.7 ± 5.5	25.7 ± 5.4	0.099
Missing	0	0	0	0	0	
Gender						0.543
Female	1	5	4	13	23	
Male	3	13	19	25	60	
Missing	0	0	0	0	0	
Weight at baseline (kg)	110.4 ± 26.2	89.8 ± 23.1	88.6 ± 25.5	83.7 ± 26.0	87.7 ± 25.5	0.223
Missing	0	0	0	0	0	
BMI at baseline (kg/m^2^)	35.0 ± 9.2	29.4 ± 7.3	28.6 ± 6.9	27.6 ± 7.2	28.6 ± 7.3	0.327
Missing	0	0	0	0	0	
Ethnic origin						0.176
Asian	1	–	3	3	7	
Black	0	2	3	1	6	
Oriental	0	1	–	1	2	
White	3	14	11	27	55	
Mixed	0	–	2	–	2	
Other	0	–	–	–	–	
Missing	0	1	4	6	11	
Education (years)	13.8 ± 2.5	13.4 ± 2.3	13.1 ± 1.7	12.9 ± 2.1	13.1 ± 2.0	0.681
Missing	0	2	3	8	13	
Number of participants	4	18	23	38	83	
Generation of antipsychotic medication at baseline						0.093
First	1	0	0	2	3	
Second	3	17	23	36	79	
First and Second	0	1	0	0	1	
Missing	0	0	0	0	0	
Generation of antipsychotic medication at follow-up						0.712
First	0	0	0	1	1	
Second	4	16	22	36	78	
First and Second	0	2	1	1	4	
Missing	0	0	0	0	0	
Baseline PANSS						
Positive	20.8 ± 5.3	16.1 ± 4.4	15.8 ± 5.2	16.3 ± 4.8	16.3 ± 4.9	0.384
Missing	0	0	0	0	0	0.966
Negative	19.0 ± 8.8	18.1 ± 6.4	16.7 ± 4.1	17.7 ± 5.7	17.5 ± 5.5	0.216
Missing	0	0	0	0	0	0.300
General	41.8 ± 12.3	35.3 ± 8.2	32.4 ± 5.6	32.7 ± 8.6	33.6 ± 8.1	
Missing	0	0	0	0	0	
Total	81.5 ± 25.8	69.5 ± 14.5	64.9 ± 10.5	66.7 ± 16.6	67.5 ± 15.3	
Missing	0	0	0	0	0	
Baseline Premorbid IQ (WTAR)	100.3 ± 12.0	101.6 ± 11.5	101.9 ± 10.8	103.3 ± 10.1	102.4 ± 10.5	0.909
Missing	0	0	0	1	1	
Baseline Current IQ (short WAIS-III)	103.3 ± 20.8	97.4 ± 14.3	95.3 ± 18.2	89.6 ± 13.4	93.6 ± 15.6	0.147
Missing	0	0	0	0	0	
Baseline CDSS	7.3 ± 6.3	6.8 ± 4.9	4.5 ± 4.0	6.1 ± 4.9	5.8 ± 4.7	0.419
Missing	0	0	0	0	0	
Baseline GAF	50.0 ± 14.1	54.3 ± 7.6	58.6 ± 8.7	56.3 ± 10.7	56.2 ± 9.8	0.305
Missing	0	0	0	0	0	

Statistical analysis of demographic and clinical variables for 83 participants with available SWATH MS proteomics data, in four follow-up weight change categories Loss >= 10 kg, Loss >= 3 kg AND < 10 kg, Within 3 kg, and Gain >= 3 kg, and the entire cohort. (Measurements of continuous variables are shown as mean ± standard deviation. P-values correspond to the statistical comparison between the distribution of variables in the four weight change categories. P-values < 0.05 indicate statistically significant differences between the compared groups.)

BMI: Body mass index; CDSS: Calgary Depression Scale for Schizophrenia; GAF: Global Assessment of Functioning; IQ: Intelligence Quotient; PANSS: Positive and Negative Syndrome Scale; SWATH MS: Sequential window acquisition of all theoretical fragment ion spectra Mass Spectrometry; WAIS: Wechsler Adult Intelligence Scale; WTAR: Wechsler Test of Adult Reading

The follow-up visit for a number of participants was conducted later than the intended time period of 12 months. For the 83 participants, this visit was conducted on average approximately 385.2 days after the Baseline visit (standard deviation = 28.0, median = 384.5, interquartile range = 27, min = 326 and max = 505 days). Weight change was divided into 4 categories: (i) participants who lost 10 kg or more, (ii) participants who lost 3 kg or more, but less than 10 kg (iii) those who remained within 3 kg of their starting weight, iv) those who increased their weight by 3 kg or more. Anything less than a 3 kg weight change over time is of questionable clinical significance in terms of weight measurement variability.

MS analysis was performed on a SCIEX 6600 TripleTOF (SCIEX, Warrington, UK). Plasma samples were prepared for MS analysis as described in [5]. The mass spectrometer method used the SWATH mode, using the 100 variable windows with optimised collision energy equations. For the LC method, buffer A was 98% Water, 2% Acetonitrile and 0.1% Formic acid with buffer B being 80% Acetonitrile, 20% Water and 0.1% Formic Acid with the total run time of 120 min. Peptides were re-suspended in a sample buffer of 4% v/v Acetonitrile and 0.1% Formic Acid. An Eksigent LC system with a nanoLC 400 autosampler and nanoLC 425 pump module were connected in line. Peptides were loaded onto a YMC-Triart C18 trap column prior to elution through a YMC-Triart C18 analytical column at 10 μl/min. Samples were run in duplicate.

Resultant mass spectrometry data files were searched through openSWATH (Version 2.0.0) [13] against a plasma library. OpenSWATH outputs were scored using PyProphet (version 0.18.3) [14] and then aligned and collated using the feature alignment script from MSproteomicstools. Downstream analysis was performed using R (version 3.4.1) [15] with the random seed set to 500. Normalisation and summarisation used the SWATH2Stats and MSstats packages from Bioconductor (release 3.5) [16]. Samples that showed an assay level FDR of 20% or greater were excluded from analysis. The application of this assay level FDR filter was primarily to remove samples where technical fault or error occurred in the acquisition. Transitions were later filtered based on m-score, as determined by PyProphet, at a threshold of 0.01.

Identifications were performed using proteotypic peptides only. At least one peptide was necessary for identification. Coefficients of variation (CVs) were calculated for each protein in each sample using the technical replicates acquired. The median of all CVs calculated was used to determine whether a sample should be reacquired in the mass spectrometer. Reacquired samples that still failed to pass the CV threshold were removed from the analysis.

### Statistics

All statistical data analyses were conducted using R (version 3.5.1) [17]. Statistical differences among the categories of weight change were analysed by Fisher’s exact test for categorical variables, one-way ANOVA test for continuous variables with normal distribution and Kruskal–Wallis test for non-parametric continuous variables. A value of p = 0.05 was used as the threshold for statistical significance.

Hierarchical clustering analysis (HCA) was performed to identify clusters of samples and proteins based on the similarity of their profiles. HCA was conducted using the hclust function in R with the distance matrix computed using the Euclidean method, and clustering using the complete linkage method. Heatmaps were plotted using the R package pheatmap.

Principal component analysis (PCA) was also conducted to explore the variation between the proteomic profiles of samples and to identify samples with possible association. PCA plots were generated using the ggbiplot R package.

The groups minocycline vs placebo as adjunctive treatment, and male vs female participants were also analysed separately. HCA and PCA were also conducted using the proteins which we previously identified as having the potential to predict future weight loss [5].

## Results

844 proteins were identified in 159 BeneMin plasma samples (these samples passed a 25% median CV cut-off filter). Following the application of a consensus filter to the data, 263 proteins were seen in 70% of these samples. HCA and PCA were conducted using the data for these 263 proteins in the 83 baseline samples corresponding to the four weight change categories.

**Fig. 1:**
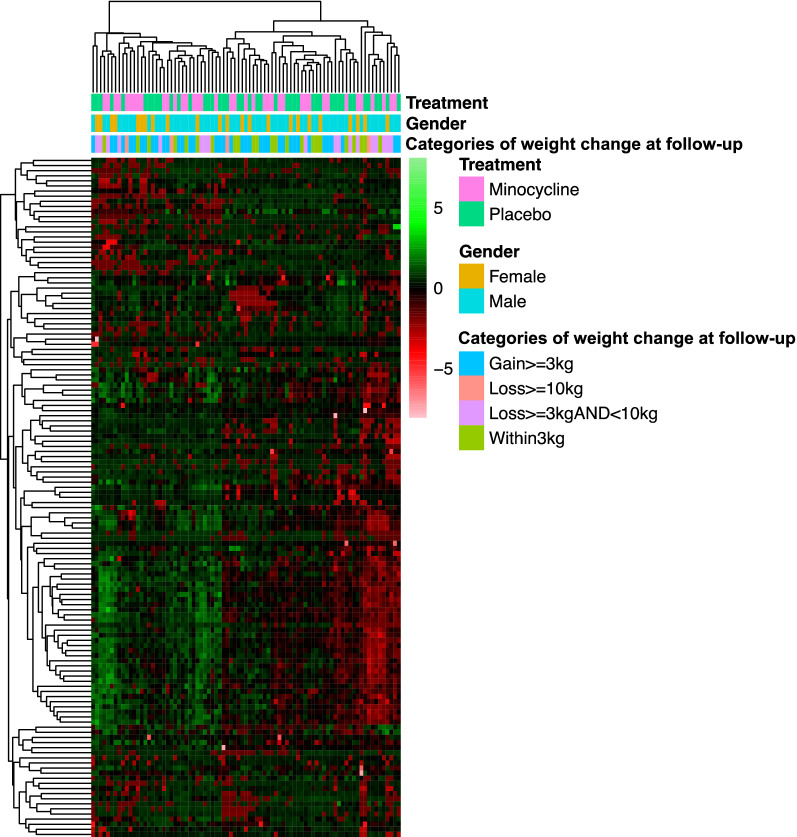
Heatmap of BeneMin baseline proteomics data for 83 participants in all four weight change categories. The dendrograms above and to the left side of the heatmap correspond to the results of hierarchical clustering analysis (HCA) for samples and proteins, respectively (the distance matrix of which was computed using the Euclidean method and clustering using the complete linkage method). The bars below the top dendrogram are coloured according to the type of adjunctive treatment (minocycline vs placebo), gender and category of weight change from baseline measured at follow-up. Heatmap was plotted using the pheatmap R package with row scaling

**Fig. 2:**
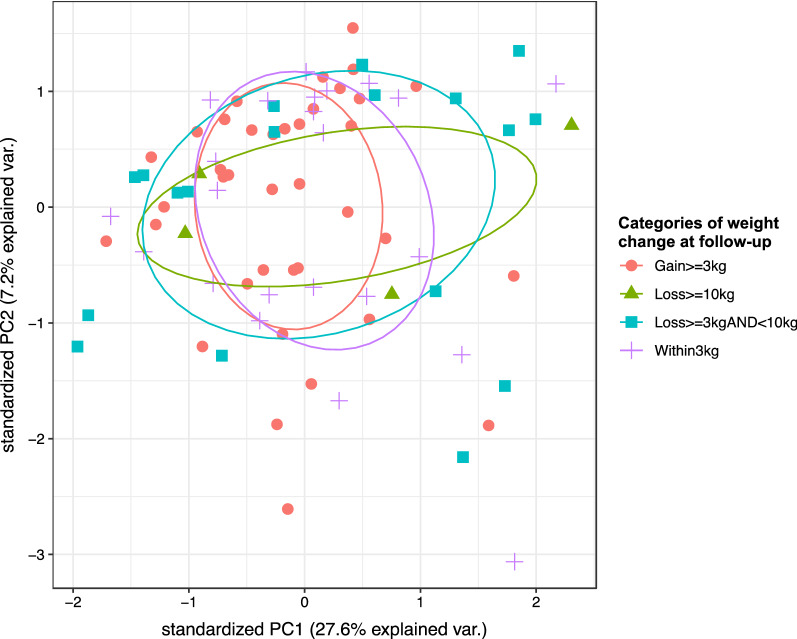
Principal component analysis (PCA) of BeneMin baseline proteomics data for 83 participants in all four weight change categories. Samples are coloured according to the category of weight change from baseline measured at follow-up. PCA was conducted for 83 participants in all four weight change categories. Results were illustrated using the ggbiplot R package

HCA and PCA of the baseline proteomics data did not reveal distinct separation between participants in different weight change categories (Figs. 1 and 2). Separate HCA in male and female participants (Additional file 1: Figure S1) and in participants who received minocycline vs placebo as adjunctive treatment (Additional file 2: Figure S2), along with separate PCA in male and female participants (Additional file 3: Figure S3) and in participants who received minocycline vs placebo (Additional file 4: Figure S4), also did not reveal clear separation in the proteomic profiles in relation to the weight change categories.

The panel of proteins that were identified in our previous study [5], also did not discriminate between the participants in different weight change categories (data not shown).

As participants in the BeneMin trial, 39/83 patients were randomised to receive minocycline and 44/83 patients were randomised to receive placebo. The allocation of minocycline and placebo in the four weight change categories was also not significantly different (p = 0.852) (Table 1).

The majority of the 83 participants were receiving SGA medication at Baseline and follow-up as part of their standard care. There was no significant difference between the four weight change categories according to whether the participants received a FGA or SGA or both at Baseline or at follow-up (p = 0.093 and p = 0.712, respectively) (Table 1). It was not possible to look at proteomic predictive value by underlying standard treatment category (SGA vs FGA), as only a small number of participants (n = 4) were taking FGA agents at Baseline.

Of the 83 participants with available baseline proteomics data, 23/83 showed weight change of ± <3 kg and 38/83 increased their weight by ≥ 3 kg, 22/83 lost 3 kg or more over the follow-up period (Table 1). The four participants who lost ≥ 10 kg, had higher positive, negative, general and total PANSS mean scores in comparison to the participants in other weight change categories, but these differences were not shown to be statistically significant (Table 1).

There were no statistically significant differences in CDSS, GAF, current IQ and premorbid IQ (Table 1).

We also further investigated the statistical differences between the four weight change categories by integrating the data for the 83 participants (with baseline proteomic profiles) with data for 26 additional BeneMin participants for whom baseline proteomic profiles were not available. Among this merged cohort of 109 participants, six individuals who lost 10 kg or more had a higher BMI at baseline (35.2 vs < 30 for other weight change categories) and had numerically higher PANSS scores, reaching statistical significance for the PANSS General score at 42.5 ± 9.9 vs 35.5 ± 7.6, 32.6 ± 5.6 and 33.6 ± 9.0 for other weight change categories (p = 0.04). None of these six individuals received metformin or aripiprazole (which has previously been reported to facilitate weight loss in patients affected by schizophrenia [18]) as part of their standard care. One of these six individuals received minocycline and five received placebo.

## Discussion

Using the technique of SWATH MS we were not able to discriminate individuals with early years psychosis who would subsequently gain weight, from those who remained weight stable or lost weight. We authors analysed proteins in this study, not lipids or metabolites, so it is possible that these non-protein entities which comprise an individual’s innate biological make-up could be determinants of weight gain in this population.

The novel nature of the BeneMin study intervention may be a factor in the failure to determine any association between the SWATH MS profile and phenotypic outcome. In particular, we saw that many individuals remained weight stable in the course of the study. Furthermore, a significant proportion of the participants in the BeneMin study were more than 12 months from first presentation, which may have influenced the results in terms of no overall relation between baseline SWATH MS profile and weight change over time. We know that much of the weight gain post-presentation with psychosis occurs in the first 3–12 months [19].

We did not show any conclusive interaction between the proteins analysed in SWATH MS and weight change. However, those individuals with the highest weight loss (they were also in the highest BMI category) had higher baseline PANSS scores. This association merits further exploration and may relate to the initially more unwell patients starting to engage with healthier lifestyle interventions as they get better and so start to lose weight. In the CATIE study there was a significant association (*p* = 0.001) between change in PANSS total score and  % change in BMI, equating to a 0.28 and 0.21 point decrease in PANSS total score (range 30–210) per 1% increase in BMI respectively [20]. However, change in BMI accounted for 3% or less of the variance of change in PANSS scores [20].

### Limitations

The number of individuals studied may have been too low in relation to the outcome measures examined here. However, this was an exploratory study to look at the application of SWATH MS in the matter of understanding what predisposes people with schizophrenia to put weight on. We anticipate that the findings may inform other studies, particularly in relation to the matter of looking at first episode psychosis individuals- as it is at this point of the patient journey that much of the weight change occurs in many people.

## Conclusion

What we have shown here is that for a research study to be able to provide a result in relation to predictors of weight change, it must be conducted in people recently diagnosed with psychosis. As it was shown before that the highest weight variation in psychosis occurs in the first 10 months on average [19, 21–23].

Our findings suggest that other factors; severity of illness, lifestyle factors and the pharmacological intervention for psychosis may be the determining factor in weight change after diagnosis, rather than any underlying proteomic factors [24] in relation to the likelihood to put on weight. This has important implications for how we can best optimise cardiometabolic outcomes in schizophrenia/schizoaffective disorder in the future.

## Supplementary information

**Additional file 1: Figure S1.** Heatmap of BeneMin baseline proteomics data for male vs female participants. Heatmap of BeneMin baseline proteomics data for (a) 60 male participants and (b) 23 female participants in all four weight change categories. The dendrograms above and to the left side of the heatmap correspond to the results of hierarchical clustering analysis (HCA) for samples and proteins, respectively (the distance matrix of which was computed using the Euclidean method and clustering using the complete linkage method). The bars below the top dendrogram are coloured according to the type of treatment (minocycline vs placebo) and the category of weight change from baseline measured at follow-up. Heatmap was plotted using the pheatmap R package with row scaling.

**Additional file 2: Figure S2.** Heatmap of BeneMin baseline proteomics data for minocycline vs placebo treatment groups. Heatmap of BeneMin baseline proteomics data for (a) 39 participants treated with minocycline and (b) 44 participants treated with placebo as adjunctive treatment, in all four weight change categories. The dendrograms above and to the left side of the heatmap correspond to the results of hierarchical clustering analysis (HCA) for samples and proteins, respectively (the distance matrix of which was computed using the Euclidean method and clustering using the complete linkage method). The bars below the top dendrogram are coloured according to gender and the category of weight change from baseline measured at follow-up. Heatmap was plotted using the pheatmap R package with row scaling.

**Additional file 3: Figure S3.** Principal component analysis (PCA) of BeneMin baseline proteomics data for male vs female participants. Principal component analysis (PCA) of BeneMin baseline proteomics data for (a) 60 male participants and (b) 23 female participants in all four weight change categories. Results were illustrated using the ggbiplot R package and are coloured according to the category of weight change from baseline measured at follow-up.

**Additional file 4: Figure S4.** Principal component analysis (PCA) of BeneMin baseline proteomics data for minocycline vs placebo treatment groups. Principal component analysis (PCA) of BeneMin baseline proteomics data for (a) 39 participants treated with minocycline and (b) 44 participants treated with placebo as adjunctive treatment, in all four weight change categories. Results were illustrated using the ggbiplot R package and are coloured according to the category of weight change from baseline measured at follow-up.

## Data Availability

Any requests for data extracts will be considered by Dr. Adrian Heald as corresponding author. Requests for sharing the anonymised trial database should be addressed to Professor Bill Deakin. The dataset is not publically available because the preparation of publications is ongoing.

## Abbreviations

BMI: Body mass index
CDSS: Calgary Depression Scale for Schizophrenia
CV: Coefficient of variation
FGA: First Generation Antipsychotic
GAF: Global Assessment of Functioning
HCA: Hierarchical clustering analysis
IQ: Intelligence Quotient
PANSS: Positive and Negative Syndrome Scale
PCA: Principal component analysis
SGA: Second Generation Antipsychotic
SMI: Severe mental illness
SWATH MS: Sequential window acquisition of all theoretical fragment ion spectra Mass Spectrometry
WAIS: Wechsler Adult Intelligence Scale
WTAR: Wechsler Test of Adult Reading
