# Supplementary figures and images for: Application of SWATH mass spectrometry in the identification of circulating proteins does not predict future weight gain in early psychosis
Source: Clin Proteomics. 2020 Oct 27;17:38. doi: 10.1186/s12014-020-09299-2 (PMC7590460; doi:10.1186/s12014-020-09299-2)

## Slide 1
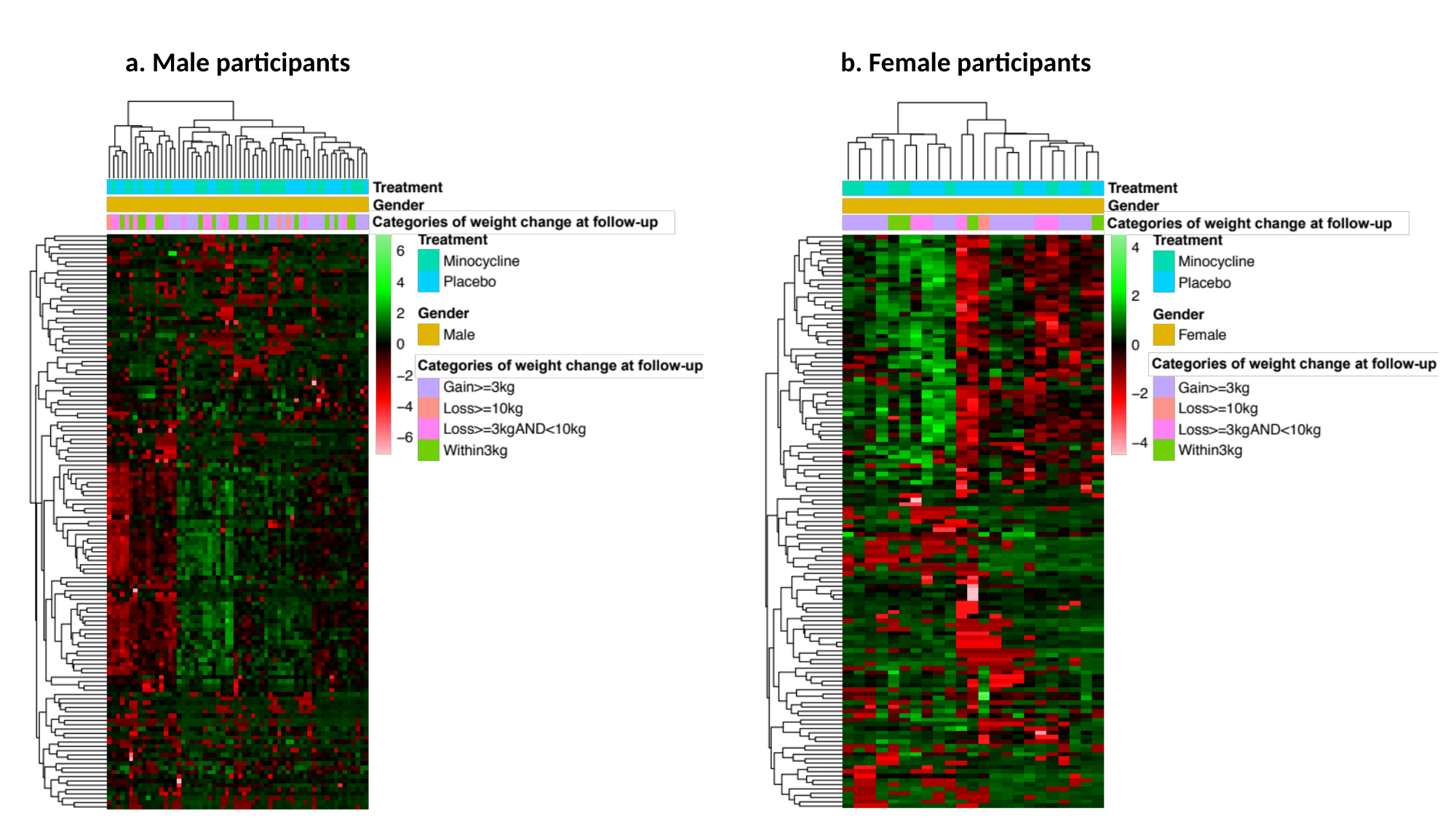

a. Male participants
b. Female participants

Supplement: Supplementary file 1 — Additional file 1: Figure S1. Heatmap of BeneMin baseline proteomics data for male vs female participants. Heatmap of BeneMin baseline proteomics data for (a) 60 male participants and (b) 23 female participants in all four weight change categories. The dendrograms above and to the left side of the heatmap correspond to the results of hierarchical clustering analysis (HCA) for samples and proteins, respectively (the distance matrix of which was computed using the Euclidean method and clustering using the complete linkage method). The bars below the top dendrogram are coloured according to the type of treatment (minocycline vs placebo) and the category of weight change from baseline measured at follow-up. Heatmap was plotted using the pheatmap R package with row scaling. [file 12014_2020_9299_MOESM1_ESM.pptx]

## Slide 1
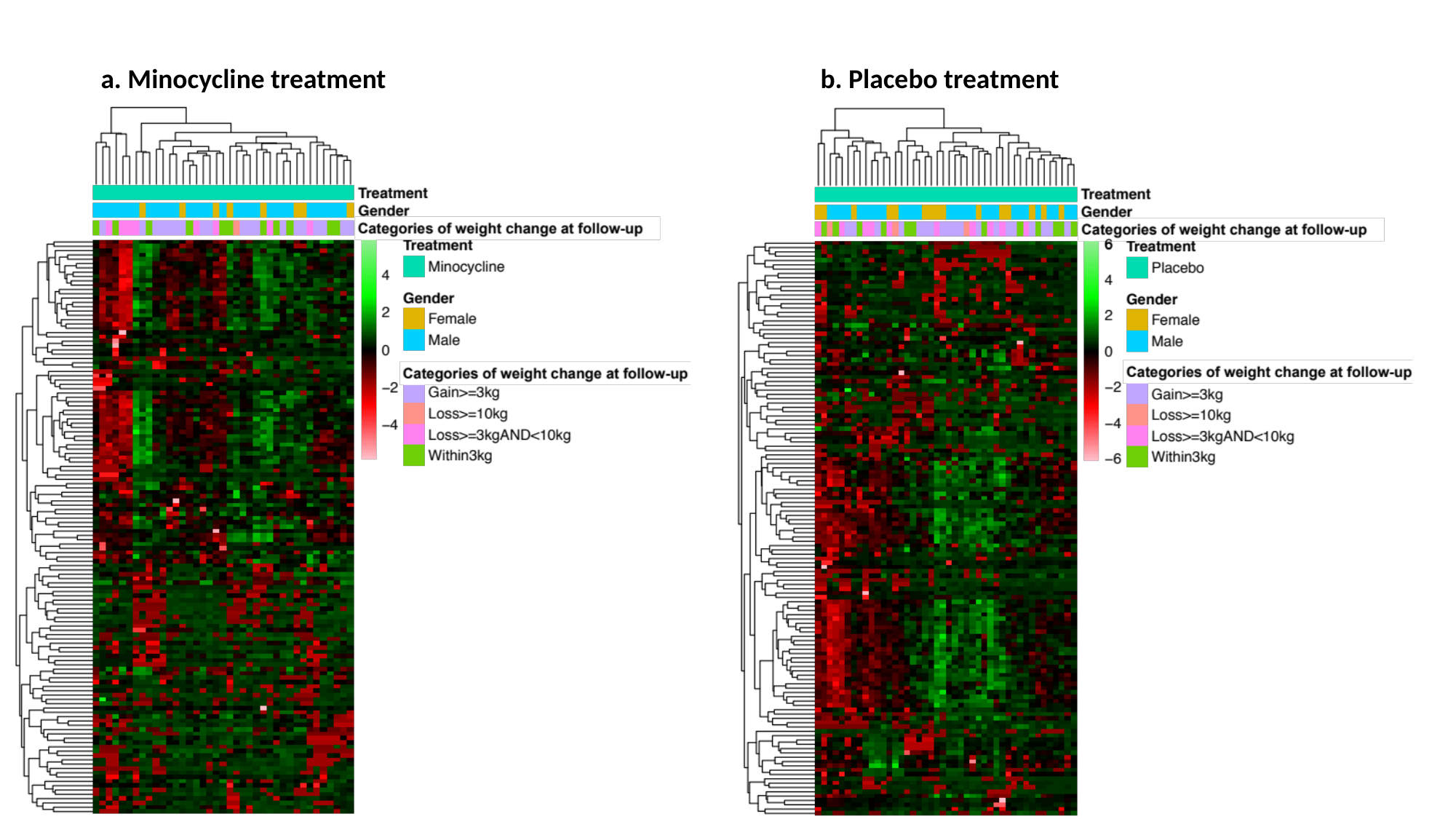

a. Minocycline treatment
b. Placebo treatment

Supplement: Supplementary file 2 — Additional file 2: Figure S2. Heatmap of BeneMin baseline proteomics data for minocycline vs placebo treatment groups. Heatmap of BeneMin baseline proteomics data for (a) 39 participants treated with minocycline and (b) 44 participants treated with placebo as adjunctive treatment, in all four weight change categories. The dendrograms above and to the left side of the heatmap correspond to the results of hierarchical clustering analysis (HCA) for samples and proteins, respectively (the distance matrix of which was computed using the Euclidean method and clustering using the complete linkage method). The bars below the top dendrogram are coloured according to gender and the category of weight change from baseline measured at follow-up. Heatmap was plotted using the pheatmap R package with row scaling. [file 12014_2020_9299_MOESM2_ESM.pptx]

## Slide 1
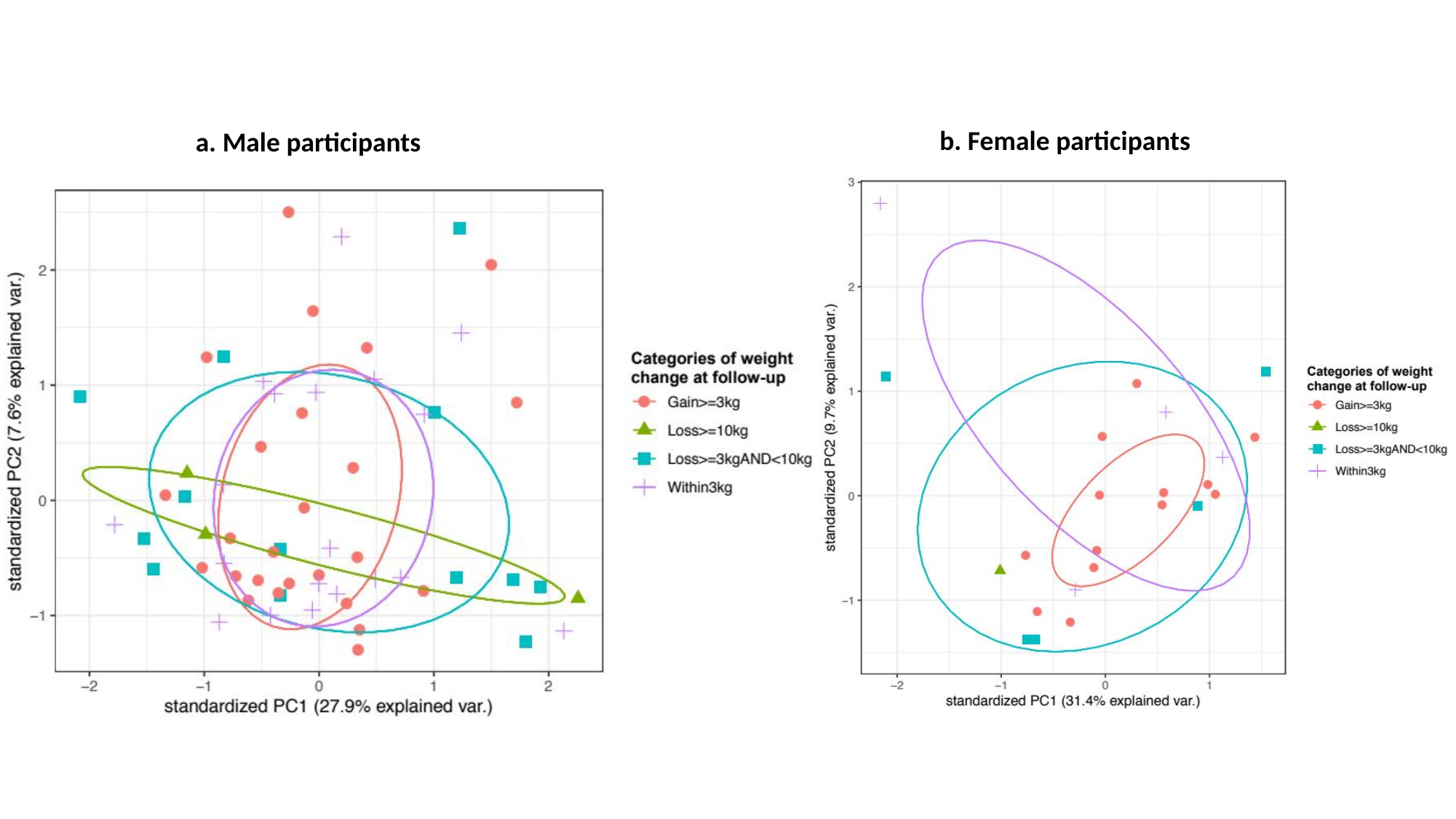

b. Female participants
a. Male participants

Supplement: Supplementary file 3 — Additional file 3: Figure S3. Principal component analysis (PCA) of BeneMin baseline proteomics data for male vs female participants. Principal component analysis (PCA) of BeneMin baseline proteomics data for (a) 60 male participants and (b) 23 female participants in all four weight change categories. Results were illustrated using the ggbiplot R package and are coloured according to the category of weight change from baseline measured at follow-up. [file 12014_2020_9299_MOESM3_ESM.pptx]

## Slide 1
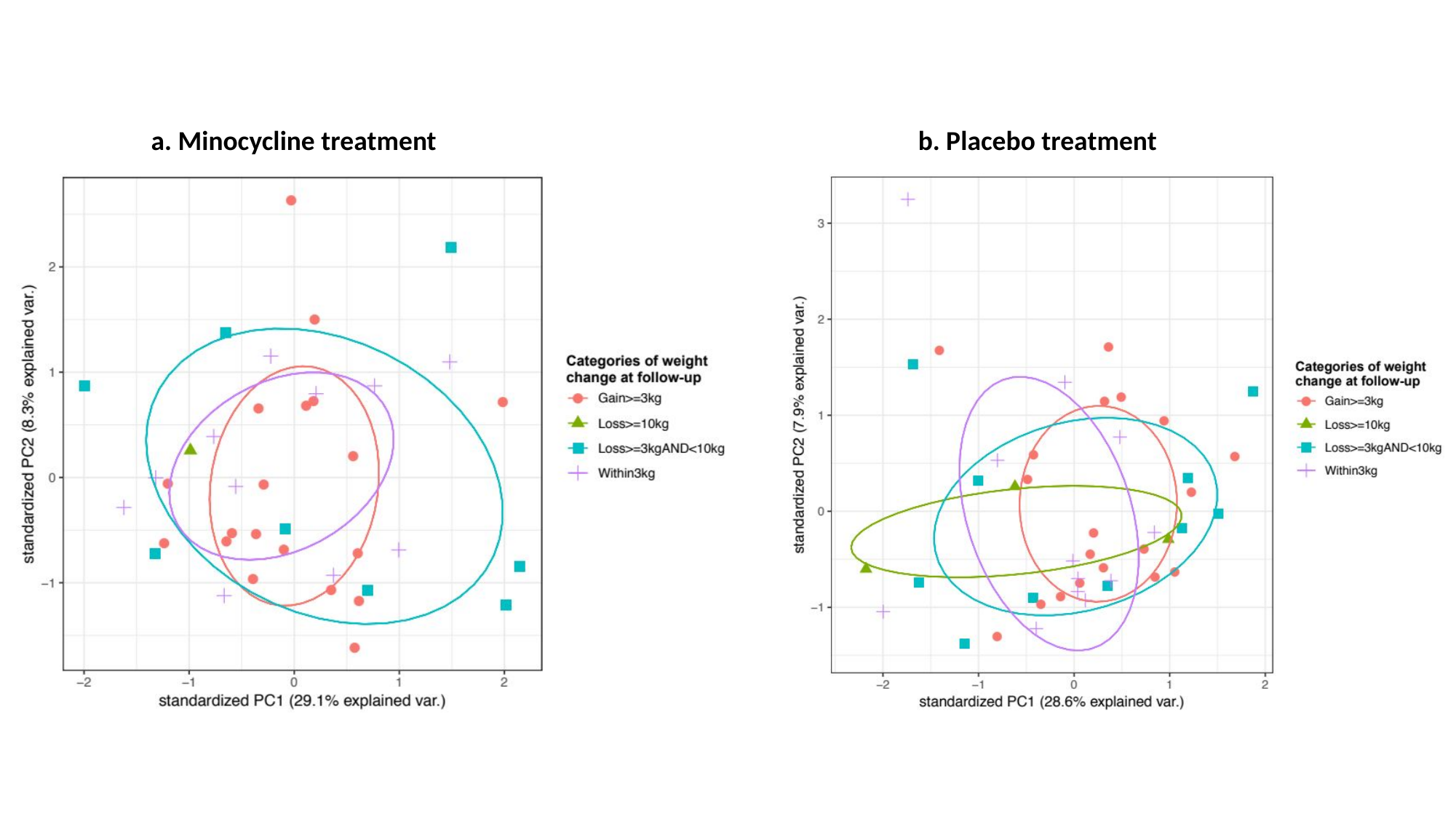

a. Minocycline treatment
b. Placebo treatment

Supplement: Supplementary file 4 — Additional file 4: Figure S4. Principal component analysis (PCA) of BeneMin baseline proteomics data for minocycline vs placebo treatment groups. Principal component analysis (PCA) of BeneMin baseline proteomics data for (a) 39 participants treated with minocycline and (b) 44 participants treated with placebo as adjunctive treatment, in all four weight change categories. Results were illustrated using the ggbiplot R package and are coloured according to the category of weight change from baseline measured at follow-up. [file 12014_2020_9299_MOESM4_ESM.pptx]
